# Hydrocyanate of Iron in Epilepsy

**Published:** 1855-01

**Authors:** 


### HYDROCYANATE OF IRON IN EPILEPSY.

The Journals from the "other side of the waters," contained a short time since notices of cases of this disease successfully treated with this therapeutic agent, and our own periodicals have republished the report. We have had for some time past a case which, although much relieved by the use of nitrate of silver, combined with tonics and antispasmodics, was not, however, entirely relieved. We thererefore determined upon a trial of this article, as it was one of those cases in which the remedy, if proper in any case, would be demanded in this. It was, however, with difficulty that the article could be found, as we had written to all those places where it was most likely it could be procured, but without success, until we addressed a note to Prof. Proctor, of the College of Pharmacy of Philadelphia, who, because it could not be found in that city, proceeded to prepare some himself, in which he succeeded, and forwarded it by mail. The patient was immediately put under its use, which is now nearly two months since, within which time he has not had a single paroxysm. His general health has improved, and his memory, which was sadly at fault, is now as retentive as before. He expresses a decided change in his feelings, and in his intercourse with family and friends is sprightly, joyful and hopeful. We indulge in the confident hope of his permanent recovery under the use of this new remedy, for which we are much indebted to Prof. Proctor, who, in his letter to us, remarked that he would refer to it in the next Journal of Pharmacy, now due, but not received.

We believe that this is the first instance in which this remedy has been used in this country—at all events it is the first of which we know any thing.

If the results in this case be permanent, the case will be reported by the father of the youth, a retired medical man, and one of the most prominent and valuable citizens of the State.

